# Supplementary material for: A validation of the Dutch version of the Awareness of Narrative Identity Questionnaire (ANIQ-NL)
Source: PLoS One. 2023 Jun 27;18(6):e0287935. doi: 10.1371/journal.pone.0287935 (PMC10298777; doi:10.1371/journal.pone.0287935)
Supplement: S1 Appendix — Dutch version of the Awareness of Narrative Identity Questionnaire. (DOCX) [file pone.0287935.s001.docx]

**S1 Appendix. ANIQ-NL.**

**Nederlandstalige versie van de Awareness of Narrative Identity Questionnaire**

Iedereen heeft herinneringen over de ervaringen die ze hebben meegemaakt gedurende hun leven. Soms kunnen deze herinneringen gebruikt worden om verhalen te creëren over ons leven. De volgende stellingen zullen je bevragen over hoe jij jouw herinneringen gebruikt om te begrijpen wat voor een persoon jij bent geweest, de persoon die je bent, en de persoon die je verwacht te worden.

Gelieve bij iedere stelling het meest toepasselijke antwoord aan te duiden.

|  | **Helemaal Helemaal**  **mee oneens mee eens** | | | | | | | | | | |
| --- | --- | --- | --- | --- | --- | --- | --- | --- | --- | --- | --- |
| 1. Mijn herinneringen zijn als verhalen die me helpen om mijn identiteit te begrijpen | 0 | 1 | 2 | 3 | 4 | 5 | 6 | 7 | 8 | 9 | 10 |
| 1. Ik gebruik mijn verhalen over mijn leven om te achterhalen wat voor een persoon ik ben | 0 | 1 | 2 | 3 | 4 | 5 | 6 | 7 | 8 | 9 | 10 |
| 1. De ervaringen uit mijn verleden vormen het verhaal over wie ik ben | 0 | 1 | 2 | 3 | 4 | 5 | 6 | 7 | 8 | 9 | 10 |
| 1. Mijn gevoel over wie ik ben, is ingebed in herinneringen aan mijn leven | 0 | 1 | 2 | 3 | 4 | 5 | 6 | 7 | 8 | 9 | 10 |
| 1. Wanneer ik nadenk over mijn leven, kan ik zien hoe er een verhaal is dat me vertelt wie ik ben | 0 | 1 | 2 | 3 | 4 | 5 | 6 | 7 | 8 | 9 | 10 |
| 1. Ik kan de gebeurtenissen van mijn leven in de volgorde plaatsen waarin ze zich voordeden | 0 | 1 | 2 | 3 | 4 | 5 | 6 | 7 | 8 | 9 | 10 |
| 1. Het is gemakkelijk voor mij om te weten in welke volgorde mijn levensgebeurtenissen zich voordeden | 0 | 1 | 2 | 3 | 4 | 5 | 6 | 7 | 8 | 9 | 10 |
| 1. Wanneer ik terugdenk aan ervaringen die ik heb meegemaakt, weet ik wanneer deze zich in mijn leven voordeden | 0 | 1 | 2 | 3 | 4 | 5 | 6 | 7 | 8 | 9 | 10 |
| 1. Ik ben me goed bewust van de volgorde waarin gebeurtenissen en ervaringen in mijn leven plaatsvonden | 0 | 1 | 2 | 3 | 4 | 5 | 6 | 7 | 8 | 9 | 10 |
| 1. Wanneer ik aan ervaringen uit mijn verleden denk, vind ik het gemakkelijk om me te herinneren wat ervoor en erna kwam | 0 | 1 | 2 | 3 | 4 | 5 | 6 | 7 | 8 | 9 | 10 |
| 1. Ik begrijp hoe het verhaal over mijn leven zich heeft ontplooid | 0 | 1 | 2 | 3 | 4 | 5 | 6 | 7 | 8 | 9 | 10 |
| 1. Ik begrijp hoe mijn levenservaringen met elkaar verbonden zijn | 0 | 1 | 2 | 3 | 4 | 5 | 6 | 7 | 8 | 9 | 10 |
| 1. Dingen die in de loop van mijn leven hebben plaatsgevonden, zijn betekenisvol aan elkaar verbonden | 0 | 1 | 2 | 3 | 4 | 5 | 6 | 7 | 8 | 9 | 10 |
| 1. Ik ben me ervan bewust hoe gebeurtenissen in mijn leven met elkaar verbonden zijn | 0 | 1 | 2 | 3 | 4 | 5 | 6 | 7 | 8 | 9 | 10 |
| 1. Ik kan begrijpen hoe ervaringen zich in mijn leven hebben voorgedaan, waarbij het ene tot het andere leidde | 0 | 1 | 2 | 3 | 4 | 5 | 6 | 7 | 8 | 9 | 10 |
| 1. Wanneer ik denk of spreek over ervaringen uit mijn verleden, kan ik thema’s zien over wat voor een persoon ik ben | 0 | 1 | 2 | 3 | 4 | 5 | 6 | 7 | 8 | 9 | 10 |
| 1. Ik kan overkoepelende thema’s over wie ik ben herkennen in de herinneringen aan mijn leven | 0 | 1 | 2 | 3 | 4 | 5 | 6 | 7 | 8 | 9 | 10 |
| 1. In de persoonlijke herinneringen aan mijn leven, merk ik thema’s op die gerelateerd zijn aan wat voor een persoon ik ben | 0 | 1 | 2 | 3 | 4 | 5 | 6 | 7 | 8 | 9 | 10 |
| 1. Wanneer ik terugdenk aan gebeurtenissen en ervaringen uit mijn leven, kan ik consistente patronen zien in de manier waarop ik denk, me voel en gedraag | 0 | 1 | 2 | 3 | 4 | 5 | 6 | 7 | 8 | 9 | 10 |
| 1. Er zijn duidelijke thema’s gerelateerd aan wie ik ben, die terug te vinden zijn in mijn persoonlijke herinneringen | 0 | 1 | 2 | 3 | 4 | 5 | 6 | 7 | 8 | 9 | 10 |
