# Supplementary material for: A validation of the Dutch version of the Awareness of Narrative Identity Questionnaire (ANIQ-NL)
Source: PLoS One. 2023 Jun 27;18(6):e0287935. doi: 10.1371/journal.pone.0287935 (PMC10298777; doi:10.1371/journal.pone.0287935)
Supplement: Attachment — Submitted filename: Review comments on PONE.docx [file pone.0287935.s002.docx]

**Review comments on PONE-D-23-09910**

**Overall comments**

The study design, data analysis, and presentation of the findings are scientifically rigorous. The outcome of the research is justifiable and it can benefit practitioners/researchers in the future. The four-factor model of Awareness of Narrative Identity Questionnaire-NL seems best fit as per the CFA parameters. The statistical procedures and data analysis process was adequately followed to confirm the validation of the tool.

**Shortcomings: areas to be improved**

- The translation of the tool and validation process was not adequately followed. From the narrative of the steps followed, a few steps such as forward translation from independent experts, consultations with experts in receiving feedback (Panel discussion with national experts on the subject), and pilot testing before the final data collection were not followed. Check more detailed procedures guided by Sousa and Rojjanasrirat (2011), and Van Ommeren et al. (1999). Such limitations are better highlighted in the limitation section so that future researchers and clinicians become aware of such caveats.
- Were there any missing data? How about the outliers?
  - It is worth mentioning the data cleaning process and exclusion criteria of any data before analysis.
- Normality tests of the data are important before applying SEM. Authors are requested to describe the assurance of normality (normal distribution) of the presented data.
- Page 21: insert Fig 1.
- As authors have used CFA rather than EFA, it is advisable to present composite reliability (i.e. construct reliability) scores with average variance extracted from each sub-scale (Wang, French, & Clay, 2015). It is also recommended to add convergent validity scores as CFA is done for validation of this tool.

I thank you for this opportunity to review this interesting paper. I wish the authors may find my comments useful to improve the quality of this paper.

***References:***

Van Ommeren, M., Sharma, B., Thapa, S., Makaju, R., Prasain, D., Bhattarai, R., &amp; de Jong, J. (1999). Preparing instruments for transcultural research: Use of the translation monitoring form with Nepali-speaking Bhutanese refugees. Transcultural Psychiatry, 36(3), 285-301. <https://doi.org/10.1177/136346159903600304>

Sousa, V. D., &amp; Rojjanasrirat, W. (2011). Translation, adaptation and validation of instruments or scales for use in cross-cultural health care research: A clear and user-friendly guideline. Journal of Evaluation in Clinical Practice, 17(2), 268–274. <https://doi.org/10.1111/j.1365-2753.2010.01434.x>

Wang, X., French, B. F., & Clay, P. F. (2015). Convergent and discriminant validity with formative measurement: A mediator perspective. *Journal of Modern Applied Statistical Methods*, *14*(1), 83-106. https://researchportal.murdoch.edu.au/esploro/outputs/journalArticle/Convergent-and-discriminant-validity-with-formative/991005544679607891
