# Supplementary material for: A validation of the Dutch version of the Awareness of Narrative Identity Questionnaire (ANIQ-NL)
Source: PLoS One. 2023 Jun 27;18(6):e0287935. doi: 10.1371/journal.pone.0287935 (PMC10298777; doi:10.1371/journal.pone.0287935)
Supplement: Attachment — Submitted filename: Response to Reviewers.docx [file pone.0287935.s003.docx]

**Response to Reviewers**

Comments

R: Replies

*M: Manuscript*

**Reviewer #1:** 
I recommend minor revision on the manuscript. I only have 2 minor suggestions that the authors may consider including in their revision.
R: Thank you for taking the time to review our manuscript. We appreciate your suggestions. We have carefully considered your comments and made revisions accordingly.

In the review section and/or in the description of the ANIQ original measure, it would be informative to include reliability values and validity assessment found in the other versions. In “Hallford and Mellor found that the ANIQ has good validity and reliability [1]. The Polish [45] and Turkish [46] translations of the ANIQ have also demonstrated good psychometric properties”, specific reliability values and validity assessment details can be included there.
R: Indeed, we have not provided detailed reliability and validity findings for the Turkish and Polish versions. We agree that it would be beneficial to include reliability/validity results found in the other translations. Including this information would provide a more complete understanding of the psychometric properties of the ANIQ in different languages. Furthermore, we have attempted to formulate more clearly that the other versions were validated by comparing their results with those of the original version (which is consistent with our approach) thus indicating consistency between the results of these translations and the original version.

*M, p. 6, Introduction: Meanwhile, the ANIQ has successfully been translated and validated in Polish [45] and Turkish [46]. The researchers conducted a psychometric comparison between the original version and the translated version to confirm their equivalence.*

*M, p. 9, Methods: Hallford and Mellor found that the ANIQ has good validity and reliability [1]. In all three studies conducted, both internal validity (α = .86-96) and test-retest reliability (r = .72-.79) were found to be high. The Polish [45] and Turkish [46] translations of the ANIQ also demonstrated favourable psychometric properties. The Polish version displayed Cronbach’s alphas ranging from .87 to .94 and test-retest coefficients between .58 and .91. As for the Turkish version, Cronbach’s alphas varied from .84 to .94, and the test-retest coefficients ranged from .77 and .95.*

Something the authors might consider addressing in the discussion, was there any previous mixed-method research that investigating autobiographical life narratives together with using this developed quantitative measure? The authors may address further about the different natures of information derived from these two approaches.

R: Yes, there is previous mixed-method research conducted that investigated autobiographical narratives together with the ANIQ. We already noted this in the introduction:

*M, p. 7, Introduction: In a third study, the authors found evidence that the ANIQ coherence subscales have criterion validity, as the scores on the subscales were significantly correlated with the more objective measure of narrative coherence provided through written turning-point narratives. Moreover, Janowicz et al. [47] found a significant correlation between the ANIQ awareness subscale and the level of coded coherence in written accounts of important interpersonal relationships.*

However, we might indeed address further about the different natures of information derived from these two approaches. We will provide additional elaboration on this in the discussion:

*M, p. 23, Discussion: As previously mentioned, Hallford and Mellor [1] found support for criterion validity by establishing correlations between the scores on the ANIQ-Coherence subscales and the coherence scores derived from written turning-point narratives (r = .23-.41). Although there is some degree of correlation between these measures, a perfect overlap is not observed. This indicates that the more objective measure of narrative coherence, involving narratives coded by an independent observer, and the more subjective measure, involving the self-report questionnaire, may not necessarily align. However, this does not necessarily suggest an inherent limitation of the questionnaire itself. Researchers utilising the ANIQ may prioritise the subjective appraisal of coherence within life stories over the actual ability to demonstrate coherence. The ability to subjectively appraise coherence within our life stories may represent another aspect of our autobiographical reasoning, other than our ability to actually demonstrate coherence in narratives. This perspective is supported by the notion that higher-order awareness of life stories is likely influenced by a range of metacognitive abilities, self-awareness, and personal agency [1]. In future studies, a combination of both measures can contribute to a more comprehensive understanding of autobiographical reasoning.*

**Reviewer #2:** 
Overall comments
The study design, data analysis, and presentation of the findings are scientifically rigorous. The outcome of the research is justifiable and it can benefit practitioners/researchers in the future. The four-factor model of Awareness of Narrative Identity Questionnaire-NL seems best fit as per the CFA parameters. The statistical procedures and data analysis process was adequately followed to confirm the validation of the tool.

Shortcomings: areas to be improved
- The translation of the tool and validation process was not adequately followed. From the narrative of the steps followed, a few steps such as forward translation from independent experts, consultations with experts in receiving feedback (Panel discussion with national experts on the subject), and pilot testing before the final data collection were not followed. Check more detailed procedures guided by Sousa and Rojjanasrirat (2011), and Van Ommeren et al. (1999). Such limitations are better highlighted in the limitation section so that future researchers and clinicians become aware of such caveats.

R: Thank you for bringing this to our attention. We appreciate your input, and upon further review, we have realized that our translation procedure could benefit from clarification. While some of the steps you mentioned were indeed included in our process, they may have been described differently. We have taken this into consideration and clarified this in the translation procedure:

*M, p. 10-11, Methods: The translation process of the original English version of the ANIQ into Dutch (ANIQ-NL) involved multiple steps. Initially, three independent translators, all native Dutch speakers, conducted a forward translation of the questionnaire from English to Dutch. One of the translators had a background in healthcare and was familiar with the terminology and construct matter in Dutch, while the other two translators were knowledgeable about informal expressions, health-care specific language, idiomatic phrases, and commonly used emotional terms in Dutch. Subsequently, the three translated versions of the instrument were compared with the support of a fourth independent translator who is an expert in the field of autobiographical memory coherence. Any ambiguities or discrepancies were discussed and resolved through a committee approach. Once the four translators reached consensus, the preliminary Dutch version was blind back-translated by experts from the Interfaculty Institute for Living Languages (ILT) at KU Leuven. Finally, a multidisciplinary committee, consisting of the four translators involved in the forward translation and the developer of the original questionnaire, David John Hallford, evaluated the translation for its accuracy. The back-translation and the original version of the questionnaire were compared to assess semantic equivalence. It turned out that the items in the Dutch version retained the intended meaning of the English items (see S1 Appendix for the final version of the ANIQ-NL).*

Although, indeed, we should acknowledge that certain steps were not included in our translation procedure, such as conducting an expert panel discussion involving national experts outside of our research department. Additionally, we did not conduct a pilot study, which could have provided additional support for the quality of our translation. These limitations will be acknowledged and discussed in the Discussion section.

*M, p. 21, Discussion: Second, it is important to acknowledge certain limitations in the translation procedure. While a rigorous translation procedure was followed, it should be noted that some steps were omitted in the process [66, 67]. For example, other national experts in the field were not involved for an expert panel discussion to gather additional feedback. Furthermore, no pilot study was conducted to assess the clarity of the instructions and items in the final version of the questionnaire. Moreover, the use of a bilingual sample could have provided further evidence for conceptual, content, and construct equivalence [66, 67].*

- Were there any missing data? How about the outliers?
– It is worth mentioning the data cleaning process and exclusion criteria of any data before analysis.

R: Prior to conducting the analyses, a data cleaning procedure was performed. Participants who provided invalid demographic information or did not complete the ANIQ-NL were excluded from the analysis (*n* = 7). Additionally, three participants completed the ANIQ-NL but not the DASS-21, and they were retained in the dataset for the psychometric analysis of the ANIQ-NL. Considering the exploratory nature of the study, outliers were retained in the dataset to ensure a rigorous examination of the psychometric properties of the ANIQ-NL. It is worth mentioning that some participants (n = 6 ) provided linear responses, resulting in exceptionally high scores on the ANIQ-NL subscales. To address this, a sensitivity analysis was conducted for these linear responses (i.e., 10-10-10-…). No differences in the results were observed. The above is integrated in the participants and data analysis description:

*M, p. 8, Methods: After a process of data cleaning (removing invalid responses, i.e., demographics invalidly entered or incomplete ANIQ-NL forms), the final sample for analysis included 541 participants between the ages of 18 and 75 (M = 34.09, SD = 15.04), of which 65.1% were female, 34.8% were male, and 0.2% identified as otherwise.*

*M, p. 13, Methods: Considering the exploratory nature of the study, outliers were retained in the dataset to ensure a rigorous examination of the psychometric properties of the ANIQ-NL. It is worth mentioning that some participants (n = 6) provided linear responses, i.e., all ANIQ-NL items were given the same answer, resulting in exceptionally high scores on the ANIQ-NL subscales. Therefore, all analyses were reconducted after removal of linear responses. No meaningful changes in the results were observed.*

- Normality tests of the data are important before applying SEM. Authors are requested to describe the assurance of normality (normal distribution) of the presented data.

R: Prior to conducting SEM and linear regression analyses, an assessment was made to determine the normality of the distribution of the results in the ANIQ-NL subscales. It was found that the distribution met the criteria for normality. This information has been included in our manuscript.

*M, p. 13, Methods: Prior to conducting data analysis, it was confirmed that the scores on the ANIQ-NL subscales followed a normal distribution, which is a prerequisite for conducting SEM and linear regression analyses.*

- Page 21: insert Fig 1.

R: Thank you for bringing this up. If we are not mistaken, according to the PLOS guidelines, figure files should be uploaded separately from the manuscript.

- As authors have used CFA rather than EFA, it is advisable to present composite reliability (i.e. construct reliability) scores with average variance extracted from each sub-scale (Wang, French, & Clay, 2015). It is also recommended to add convergent validity scores as CFA is done for validation of this tool.

R: Thank you for noting this. We added the average variance extracted (AVE) scores for each subscale as an extra measure of convergent validity, besides fit indices (CFI, RMSEA, SRMR), factor loadings and intercorrelations among the factors. Moreover, we added composite reliability (CR) scores as a more accurate measure of reliability in the context of structural equation modelling. Although, we kept the Cronbach’s alphas as an additional measure of reliability for comparison with the original validation study (and the Turkish and Polish validation studies).

*M, p. 12, Methods: Cronbach’s alphas and composite reliability scores (CR) were used to assess the internal consistency of the subscales. In addition to Cronbach’s alpha, CR is a measure that takes into account the factor loadings of the items and allows for correlated errors [54-56]. The following rules of thumb are used to interpret Cronbach’s alphas: < .50 is unacceptable, ≥ .50 is poor, > .60 is questionable, > .70 is acceptable, > .80 is good, and > .90 is excellent [57]. Similarly, CR values above 0.70 are generally considered acceptable [54]. To evaluate the convergent validity of the ANIQ-NL subscales, average variance extracted (AVE) scores were computed. AVE represents the proportion of variance captured by the latent factor in relation to the measurement error. AVE scores of 0.50 or higher are typically considered acceptable [54].*

*M, p. 15, Results: The CR scores and AVEs were added in Table 2.*

|  | Awareness | Temporal Coherence | Causal Coherence | Thematic Coherence | Total |
| --- | --- | --- | --- | --- | --- |
| AVE | .50 | .81 | .68 | .73 | .65 |
| CR | .81 | .99 | .99 | .99 | .96 |

*M, p. 16, Results: The CR scores provided additional support for a high level of internal consistency, with values ranging from .81 to .99. This suggests that the items within each subscale consistently and reliably measured the underlying construct. Furthermore, the AVE scores for all subscales exceeded the recommended threshold of 0.50, indicating that the items accounted for a substantial proportion of the variance within their respective constructs.*

*M, p. 19, Discussion: In addition, the ANIQ subscales were found to have good to excellent internal consistency. Across all subscales, the items were able to explain a favourable amount of variance within their respective subscales.*

I thank you for this opportunity to review this interesting paper. I wish the authors may find my comments useful to improve the quality of this paper.

R: Thank you for taking the time to review our manuscript. We appreciate your suggestions. We have carefully considered your comments and made revisions accordingly.

References:
Van Ommeren, M., Sharma, B., Thapa, S., Makaju, R., Prasain, D., Bhattarai, R., & de Jong, J. (1999). Preparing instruments for transcultural research: Use of the translation monitoring form with Nepali-speaking Bhutanese refugees. Transcultural Psychiatry, 36(3), 285-301. <https://doi.org/10.1177/136346159903600304>
Sousa, V. D., & Rojjanasrirat, W. (2011). Translation, adaptation and validation of instruments or scales for use in cross-cultural health care research: A clear and user-friendly guideline. Journal of Evaluation in Clinical Practice, 17(2), 268–274. <https://doi.org/10.1111/j.1365-2753.2010.01434.x>
Wang, X., French, B. F., & Clay, P. F. (2015). Convergent and discriminant validity with formative measurement: A mediator perspective. Journal of Modern Applied Statistical Methods, 14(1), 83-106. <https://researchportal.murdoch.edu.au/esploro/outputs/journalArticle/Convergent-and-discriminant-validity-with-formative/991005544679607891>
